# Activation of biceps femoris long head reduces tibiofemoral anterior shear force and tibial internal rotation torque in healthy subjects

**DOI:** 10.1371/journal.pone.0190672

**Published:** 2018-01-05

**Authors:** Nur Liyana Azmi, Ziyun Ding, Rui Xu, Anthony M. J. Bull

**Affiliations:** 1 Department of Bioengineering, Imperial College London, London, United Kingdom; 2 Department of Biomedical Engineering, Tianjin University, Tianjin, China; Universite de Nantes, FRANCE

## Abstract

The anterior cruciate ligament (ACL) provides resistance to tibial internal rotation torque and anterior shear at the knee. ACL deficiency results in knee instability. Optimisation of muscle contraction through functional electrical stimulation (FES) offers the prospect of mitigating the destabilising effects of ACL deficiency. The hypothesis of this study is that activation of the biceps femoris long head (BFLH) reduces the tibial internal rotation torque and the anterior shear force at the knee. Gait data of twelve healthy subjects were measured with and without the application of FES and taken as inputs to a computational musculoskeletal model. The model was used to investigate the optimum levels of BFLH activation during FES gait in reducing the anterior shear force to zero. This study found that FES significantly reduced the tibial internal rotation torque at the knee during the stance phase of gait (p = 0.0322) and the computational musculoskeletal modelling revealed that a mean BFLH activation of 20.8% (±8.4%) could reduce the anterior shear force to zero. At the time frame when the anterior shear force was zero, the internal rotation torque was reduced by 0.023 ± 0.0167 Nm/BW, with a mean 188% reduction across subjects (p = 0.0002). In conclusion, activation of the BFLH is able to reduce the tibial internal rotation torque and the anterior shear force at the knee in healthy control subjects. This should be tested on ACL deficient subject to consider its effect in mitigating instability due to ligament deficiency. In future clinical practice, activating the BFLH may be used to protect ACL reconstructions during post-operative rehabilitation, assist with residual instabilities post reconstruction, and reduce the need for ACL reconstruction surgery in some cases.

## Introduction

Healthy loading of the tibiofemoral joint of the knee during activities of daily living including gait involves significant tibial anterior shear and tibial internal rotation torque [1]. The anterior cruciate ligament (ACL) is the primary restraint to anterior shear and a major secondary restraint to internal tibial rotation [2]. Therefore, ACL deficiency through sports trauma results in anterior tibial translation and tibial internal rotational instability of the knee [3].

Knee movement is a function of external forces and of muscle forces [4]. In ACL-deficiency, knee joint stability is provided through the action of concavity compression of the tibiofemoral articulation on the medial side, where the compressive forces push together the concave surface of the joint. However, this stability mechanism is not present at the lateral knee compartment as the lateral tibial plateau is convex, resulting in an unstable and more mobile compartment. As a result, during normal knee joint loading with a tibial rotational torque, the rotational axis of the knee moves medially, creating an excessive translation of the lateral compartment [5–8]. This excessive movement then causes secondary conditions including damage to the other passive restraints to these motions, such as cartilage, menisci, and the collateral ligaments [9–11]. ACL deficiency is implicated with an increase in the rate of osteoarthritis [12, 13] and limits athletes in their activity [14].

There is a subset of ACL deficient patients who are able to return to pre-injury activity without surgical intervention; these are termed copers. Coping is achieved through avoiding muscular contraction that produces an anterior shear force through, for example, avoiding full contraction of the quadriceps especially during the early stance phase and when the knee is at full extension [15]. An alternative coping mechanism counteracts quadriceps contraction through co-contraction of the hamstrings [16, 17] and through the adaptation of muscle firing [18]. The other set is that of non-copers who undergo ACL reconstruction surgery [16], where there may be a residual internal rotation instability [19]. Prior work has shown that activating muscles crossing the knee with functional electrical stimulation (FES) is able to reduce anterior tibial translation (ATT), a surrogate measure of the anterior shear force [20]. It has also been shown that FES, assisted with a knee brace, can be used to learn a muscle contraction pattern that then, once learned, persists despite halting the use of FES [12]. Thus, the underpinning hypothesis of this work is that FES can restore normal ATT at the lateral compartment of the knee by entraining the contraction of specific knee muscles.

The main muscles involved in the movement of the knee are the quadriceps, gastrocnemius and hamstrings. Of these, the hamstrings afford the most potential to reduce anterior tibial shear force and thus restore ATT to normal [4, 21, 22] as they are anatomically located to apply a posterior pull to the tibia [23]. Biceps femoris long head (BFLH) is the best candidate for selective activation in order to resist the peaks of anterior shear force and internal rotation torques during the stance phase of gait [4]. It has been shown in a modelling study that activation of BF is able to decrease the anterior tibial shear force when knee flexion is less than 40° [24]. Additionally, because BFLH attaches to the fibular head on the lateral aspect of the knee, it is expected that it will also be able to resist the large internal rotation torque and hence the large pathological motion of the lateral compartment in ACL deficiency [5, 6]. Thus, it is hypothesized that activation of BFLH is able to restore knee stability in non-copers to allow them to become copers.

This preliminary study addresses two main questions: first, can muscle activation reduce the internal rotation torque and the anterior tibial shear force? Secondly, what is the optimum muscle activation to achieve this? Also, the effects of the muscle activation on other loading across the knee is examined. The study is a combination of computational modelling and an *in vivo* experimental study in healthy control subjects.

## Materials and methods

### Physical experiments

**Fig 1:**
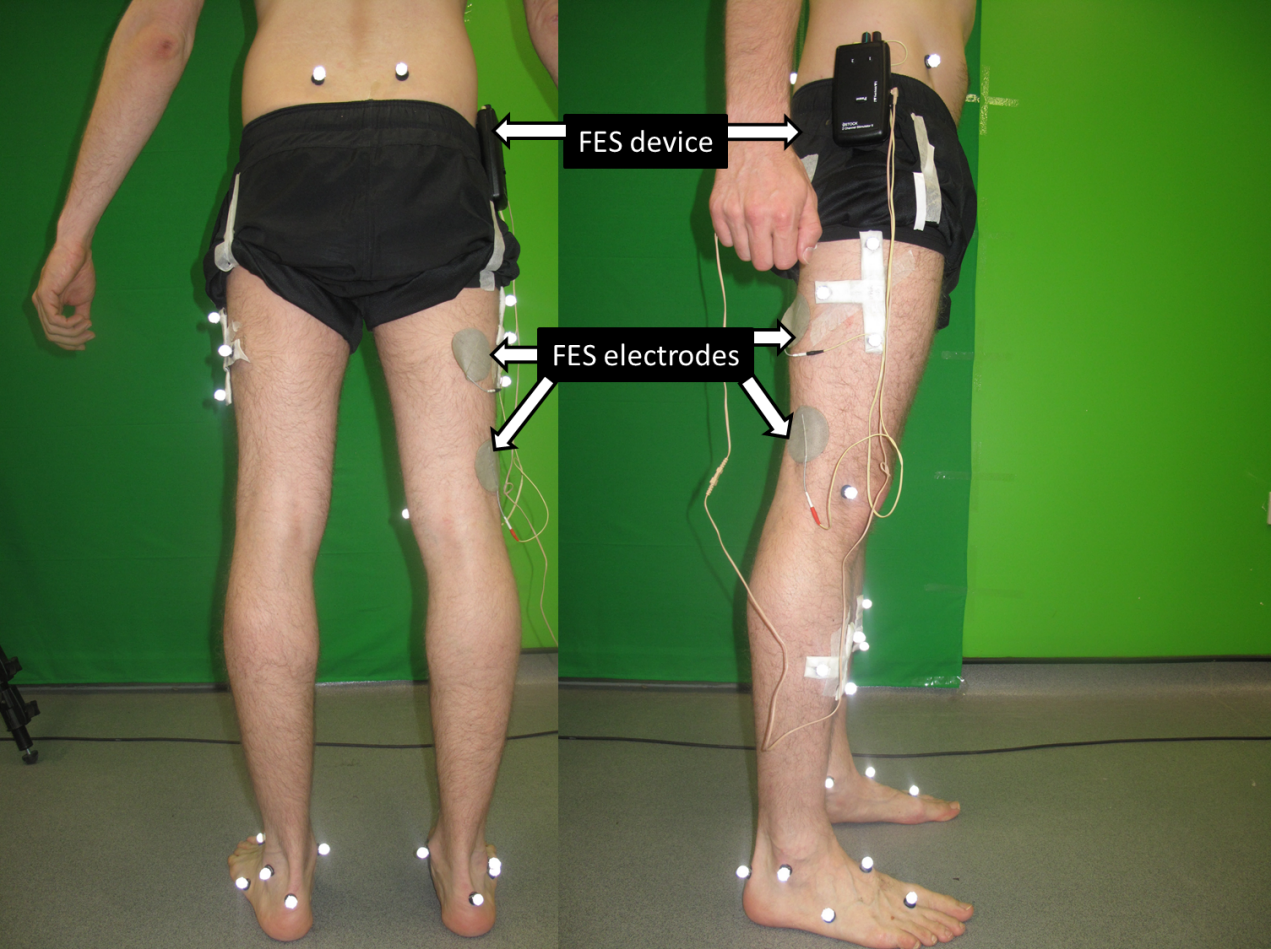
Optical motion tracking markers and FES electrodes positioning.

This study was approved by the Imperial College London Research Ethics Committee and written informed consent was obtained from all participants. Twelve healthy subjects (5 male, 7 female; height 1.67 ± 0.08 m; mass 66.74 ± 16.80 kg; age 26.08 ± 2.29 years) were recruited and underwent normal gait and functional electrical stimulation (FES) gait in sequence. During normal gait, subjects walked across the walkway in a self-selective comfortable walking speed, taking several steps prior to landing the right foot entirely on the force plate, and continuing for several steps. FES gait immediately followed the normal gait. The skin of the right BFLH region was prepared with 70% isopropyl alcohol skin wipes and two FES electrodes (Odstock 2 Channel Stimulator, Odstock Medical Ltd., UK) were placed over the region: one placed in the middle of the line between the ischial tuberosity and the lateral epicondyle of the tibia, and the other placed two hand widths distal to the first [25]. The frequency of the stimulator was set to the manufacturer recommended level of 40 Hz and simulation current was set to a minimum value of 40 mA. The subject was asked to stand on their left leg in a neutral position, while the stimulator was activated. In all cases this resulted in visible flexion of the right knee, confirming the excitation of BFLH. The intensity was then adjusted to the maximum level that the subject was able to comfortably withstand. The FES stimulation current was set up to start with one second of ramp up, followed by four seconds of maximum current and then one second of ramp down. The stimulator was manually started by the subject and timed so that the stimulation current was at its maximum value from when the right foot stepped on the force plate, through heel strike, until toe off. Several practice trials were made to allow the subject to become accustomed to the required timing after which motion capture commenced. Ground reaction forces (GRF) were recorded at 1000 Hz from a force plate (Kistler Type 9286BA, Kistler Instrument AG, Winterthur, Switzerland). A ten-camera motion analysis system (Vicon Motion Systems Ltd, Oxford, UK) recorded the motion of the right lower limb at 200 Hz; eighteen retro-reflective markers were attached to the foot, thigh and pelvis with an additional two clusters of three markers attached to the shank and thigh [26] (Fig 1). The subjects walked for six trials for normal gait and six for FES gait, of which a random selection of three trials each were used for data analysis.

### Lower limb musculoskeletal model

Open source musculoskeletal modelling software, Freebody V2.1 [27, 28], was used. This has been validated for knee joint forces and muscle activity using direct measures from instrumented implants and electromyography [28]. The segment-based lower limb model consists of the foot, shank, thigh, pelvis and patella (the patella segment is assumed to be massless, and its position and orientation is determined based on the knee flexion angles and the geometry of the patellofemoral joint [27]). The model inputs are the kinematics data from the retro reflective markers and the kinetic data from the force plate. The model calculates the intersegmental forces and torque at the proximal end of each segment [29]. Each subject’s anatomical geometry was created by linear scaling of an MRI-based anatomical dataset. The dataset consists of 163 muscle elements representing 38 lower limb muscles. The muscle attachment sites, joint centres of rotation, and tibiofemoral contact points were manually digitized from the MR imaging of a male subject (1.83 m, 96 kg, 44 years) [28]. The model quantifies the muscular and joint reaction forces experienced by the lower limb during the recorded movement through minimisation of a cost function [30]:

$$min{\sum_{i=1}^{163}\left(\frac{f_{i}}{f_{max_{i}}}\right)}^{3}$$ (1)

where *f*_*i*_ is the muscle force of muscle element *i* (*i* = 1,…,163) and *f*_*maxi*_ is the maximal muscle force of muscle element *i*, which is determined by multiplying published physiological cross-sectional areas of muscle element *i* by an assumed maximum muscle stress of 31.39 N/cm^2^ [31], constrained by the equations of motion of the whole lower limb:

$$\left[\begin{array}{c} S_{i}\\ M_{i} \end{array}\right]=\left[\begin{array}{cc} m_{i}E_{3\times 3} & 0_{3\times 3}\\ m_{i}c_{i} & I_{i} \end{array}\right]\;\left[\begin{array}{c} a_{i}-g\\ \overset{¨}{\theta_{i}} \end{array}\right]+\left[\begin{array}{c} 0_{3\times 1}\\ {\dot{\theta }}_{i}\times I_{i}{\dot{\theta }}_{i} \end{array}\right]\;\left[\begin{array}{cc} E_{3\times 3} & 0_{3\times 3}\\ d_{i} & E_{3\times 3} \end{array}\right]\;\left[\begin{array}{c} S_{i-1}\\ M_{i-1} \end{array}\right]$$ (2)

where *i* is the segment number or joint number (numbering from distal to proximal), ***S_i_*** the proximal intersegmental forces, ***S***_***i*−1**_ the distal inter-segmental forces, ***M_i_*** the proximal intersegmental torques (notional joint torques), ***M***_***i*−1**_ the distal intersegmental torques (notional joint torques), ***I_i_*** the inertia tensor, ${\overset{¨}{\theta }}_{i}$ the angular acceleration about COM, ***a_i_*** the linear acceleration of COM, *m*_*i*_ the segment mass, *E*_3×3_ the identity matrix, ***c_i_*** the vector from the proximal joint to the segment COM and ***d_i_*** is the vector from the proximal to the distal joint.

In order to quantify the effect of higher muscle activation of BFLH produced by the FES at the knee, a revised optimisation method is proposed:

$$min{\sum_{i=1}^{162}\left(\frac{f_{i}}{f_{max_{i}}}\right)}^{3}$$ (3)

where *f*_*i*_ is the muscle force of muscle element *i* (*i* = 1,…,162) and $f_{max_{i}}$ is the maximal muscle force of muscle element *i*.

In the revised optimisation method, the muscle force of BFLH is set as a constant value during the stance phase to replicate the physical stimulation of the muscle by FES. This value is set at a muscle activation, *c* times the maximum force of BFLH. As the attachment sites of BFLH are on the shank and thigh segments, the equations of motion of the shank and thigh segments were modified by the inclusion of an additional term to give:

$$\left[\begin{array}{c} S_{i}\\ M_{i} \end{array}\right]=\left[\begin{array}{cc} m_{i}E_{3\times 3} & 0_{3\times 3}\\ m_{i}c_{i} & I_{i} \end{array}\right]\;\left[\begin{array}{c} a_{i}-g\\ \overset{¨}{\theta_{i}} \end{array}\right]+\left[\begin{array}{c} 0_{3\times 1}\\ \dot{\theta_{i}}\times I_{i}\dot{\theta_{i}} \end{array}\right]+\left[\begin{array}{cc} E_{3\times 3} & 0_{3\times 3}\\ d_{i} & E_{3\times 3} \end{array}\right]\;\left[\begin{array}{c} S_{i-1}\\ M_{i-1} \end{array}\right]-\left[\begin{array}{c} \left(c\times f_{BFLH_{max}}\right)\cdot n_{BFLH}\\ \left(c\times f_{BFLH_{max}}\right)\cdot (r_{BFLH}\times n_{BFLH}) \end{array}\right]$$ (4)

where *c* is a constant, $f_{{BFLH}_{max}}$ the maximum force of BFLH, ***n***_***BFLH***_ the line of action of BFLH and ***r***_***BFLH***_ the moment arm of BFLH. In this study, *c* was increased in increments of 0.05 until the peak anterior tibial shear was reduced to zero, where *c* is a value between 0 and 1, to make sure that the BFLH force does not exceed its maximum activation value. The reduction in BFLH activation theoretically causes a reduction in tibial internal torque, which was calculated as the product of the reduction of BFLH muscle force and its moment arm at the time frame at which peak anterior tibial shear occurred.

### Data analysis

The stance phase was expressed in a 0–100% duration with a step interval of 1% using cubic spline data interpolation. Walking speed, knee joint torque, anterior shear force, knee contact force and patella tendon force were measures of interest and expressed as the mean value of three trials. Knee joint torque and knee contact force were presented in the tibial coordinate frame. Patella tendon force was calculated from the force balance across the patellofemoral joint. To test the hypothesis that the peak of the tibial internal rotation torque and the anterior shear force were reduced by applying the FES over the BFLH, the differences between normal gait and FES gait were compared using a one-tail paired-samples t-test with an α level of 0.05. All data processing and analysis was conducted in MATLAB (The Mathworks Inc., Natick, MA).

## Results

Walking speed during FES gait (0.25m/s) was significantly reduced by 7% compared to normal gait (0.27m/s; *p* = 0.036).

**Fig 2:**
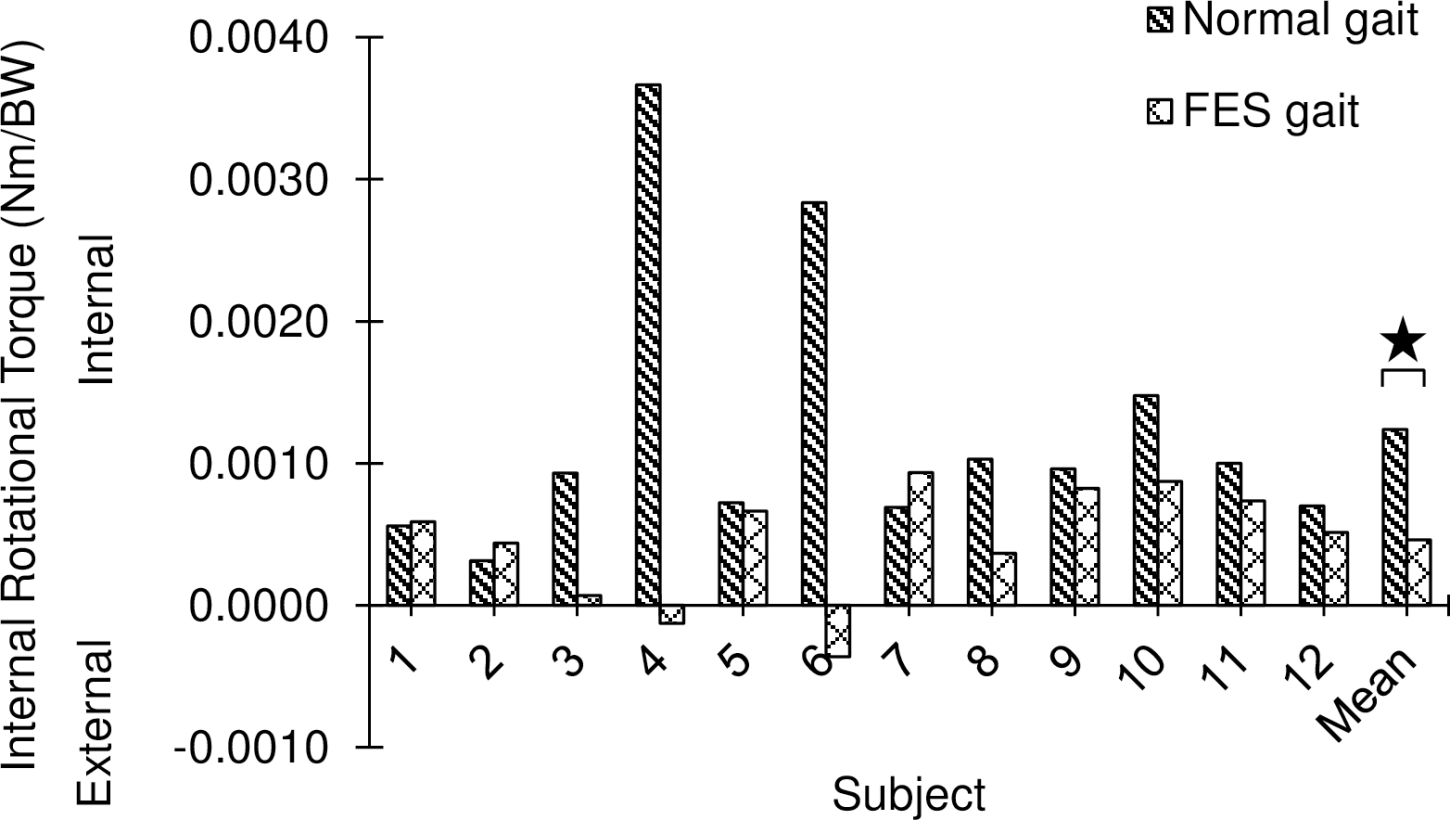
Peak knee internal rotation torque across twelve subjects during normal and FES gait.

**Fig 3:**
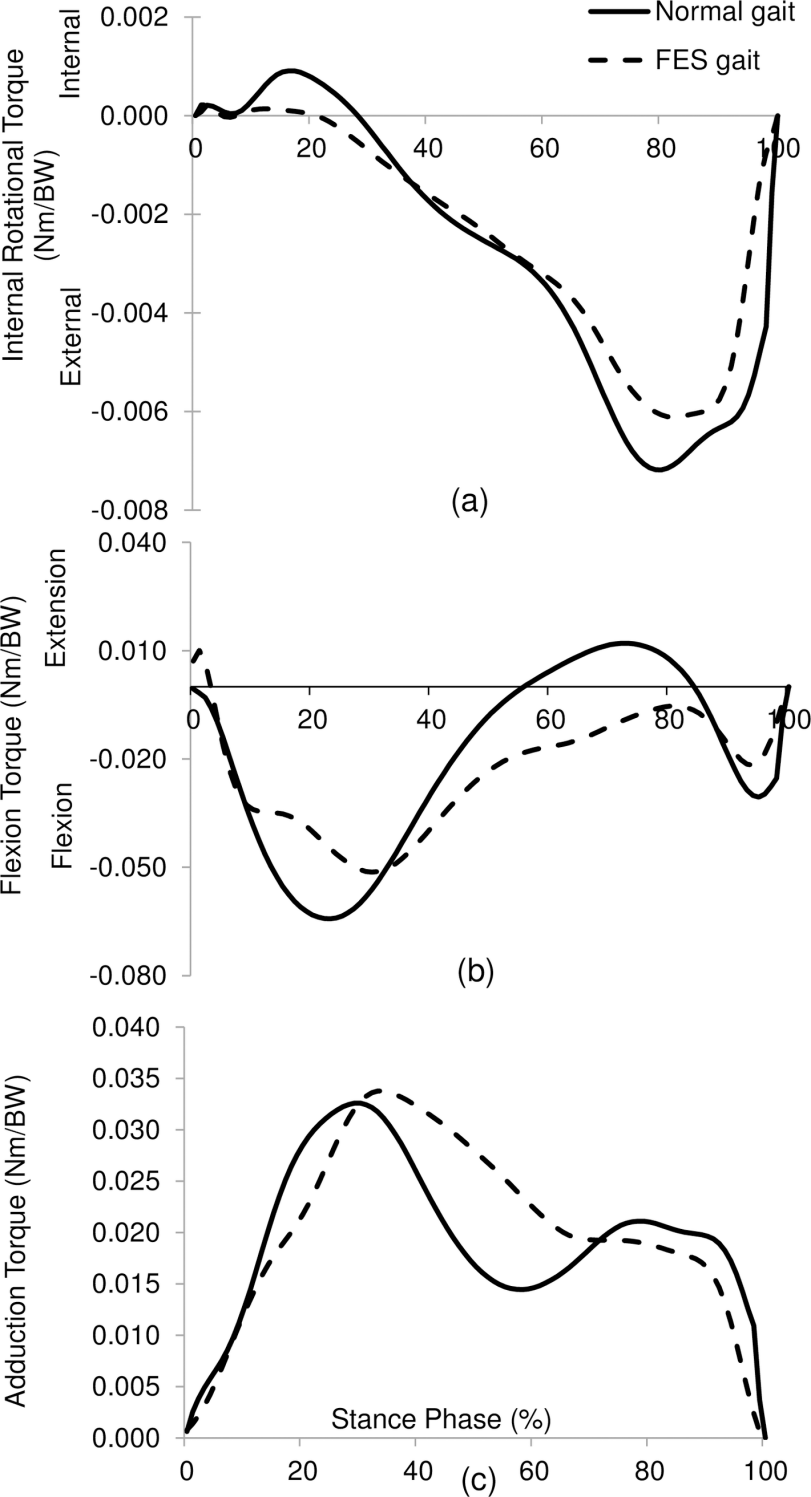
Knee joint torques in three planes for one representative subject during normal and FES gait (mass 48.9kg, height 1.60m): (a) transverse, (b) sagittal, and (c) frontal planes.

The peak value of the tibial internal rotation torque across all subjects was 0.0012± 0.0010 Nm/BW during normal gait. It was reduced by 63% to 0.0005 ±0.0004 Nm/BW (*p* = 0.032) when BFLH was stimulated by FES (Fig 2). The first peak of adduction torque and of flexion torque were not significantly different during FES gait compared to normal gait (*p* = 0.3457 and *p* = 0.2623, respectively; Fig 3).

**Fig 4:**
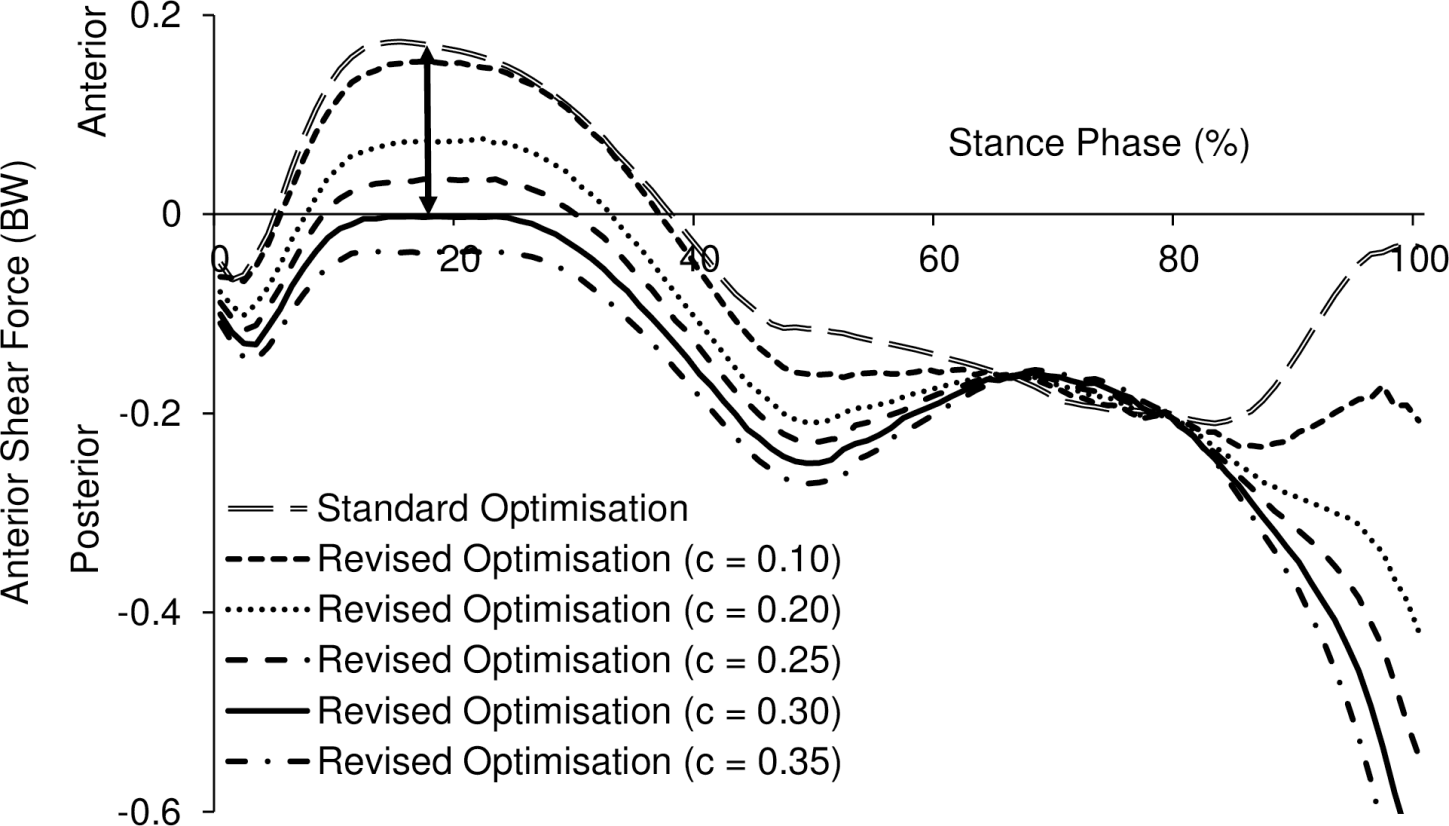
Anterior shear force for one representative subject (mass 48.9kg, height 1.60m) using standard and revised optimisation methods with different values of *c*. The activation of biceps femoris long head is expressed as a *c* value. The arrow indicates that the peak anterior shear force was reduced to zero with *c* = 0.30 in the revised optimisation.

**Fig 5:**
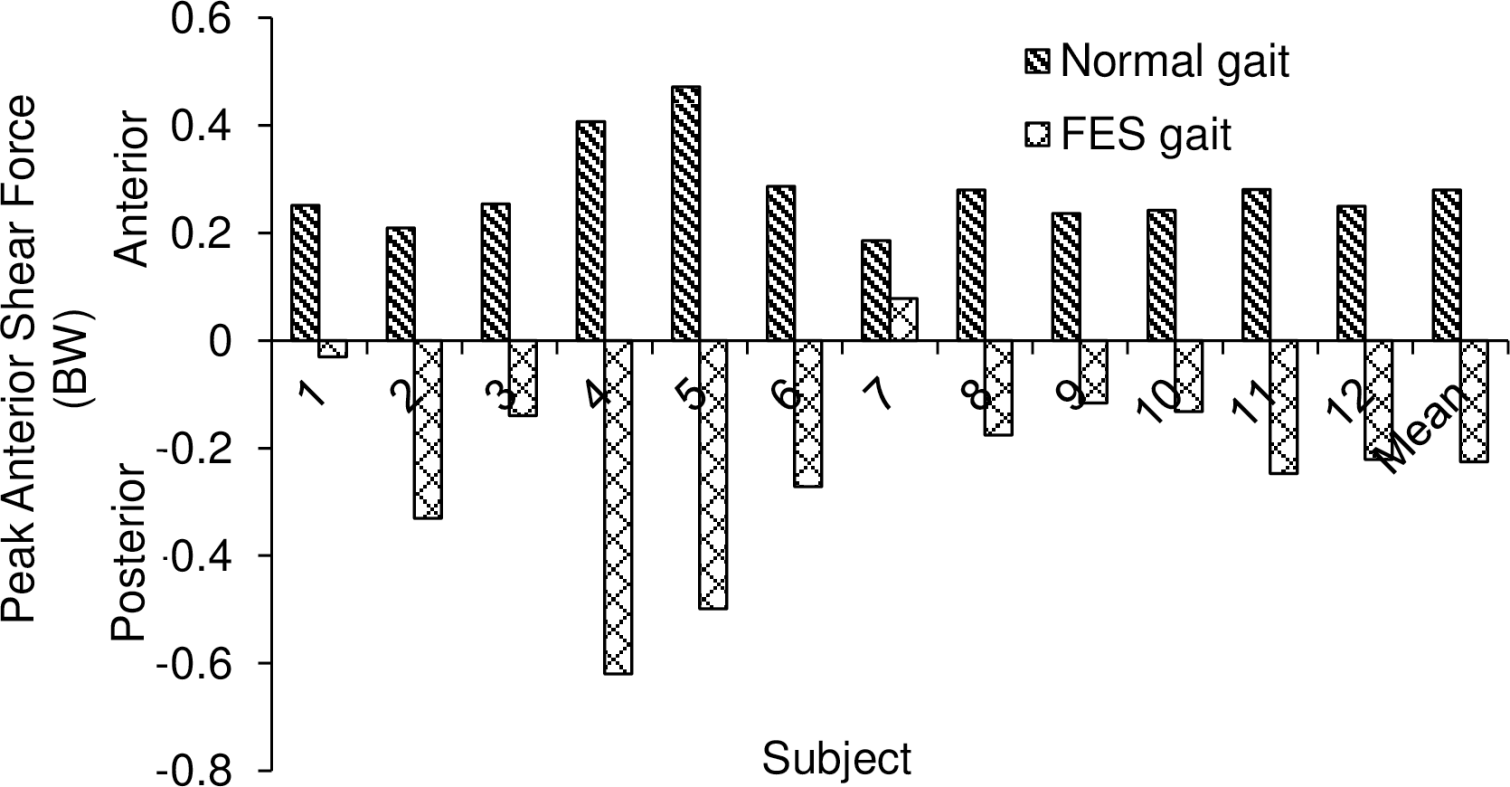
Peak anterior shear force using standard and revised optimisation methods (n = 12, *c* = 0.208).

In the standard optimisation method, the peaks of anterior shear force and internal rotation torque both occurred at late loading response (between 12.6% and 18.8% of the stance phase). Mean peak anterior shear was 0.2892 ± 0.0766 BW. The muscle activation of BFLH at peak anterior shear was 0.0152 ± 0.0214. Increasing BFLH activation incrementally resulted in an incremental reduction in the anterior shear force (Fig 4). The activation of BFLH (expressed as a *c* value) required to reduce the peak anterior shear force to zero in the revised optimisation ranged from 0.15 to 0.40 with a mean *c* value of 0.208 ± 0.084. Applying the mean value of 0.208 to all subjects, reduced the peak anterior shear force to below zero in 11/12 subjects and was 0.0778 BW for the other subject (Fig 5). At the time frame at which peak anterior shear force occurred, the reduction in tibial internal torque was calculated as the product of the reduction of BFLH muscle force and its moment arm at that time frame and normalised by body weight. This level of muscle activation at 0.208 caused a 188% reduction of the internal rotational torque of 0.0226 ± 0.0167 Nm/BW (*p* = 0.0002).

**Fig 6:**
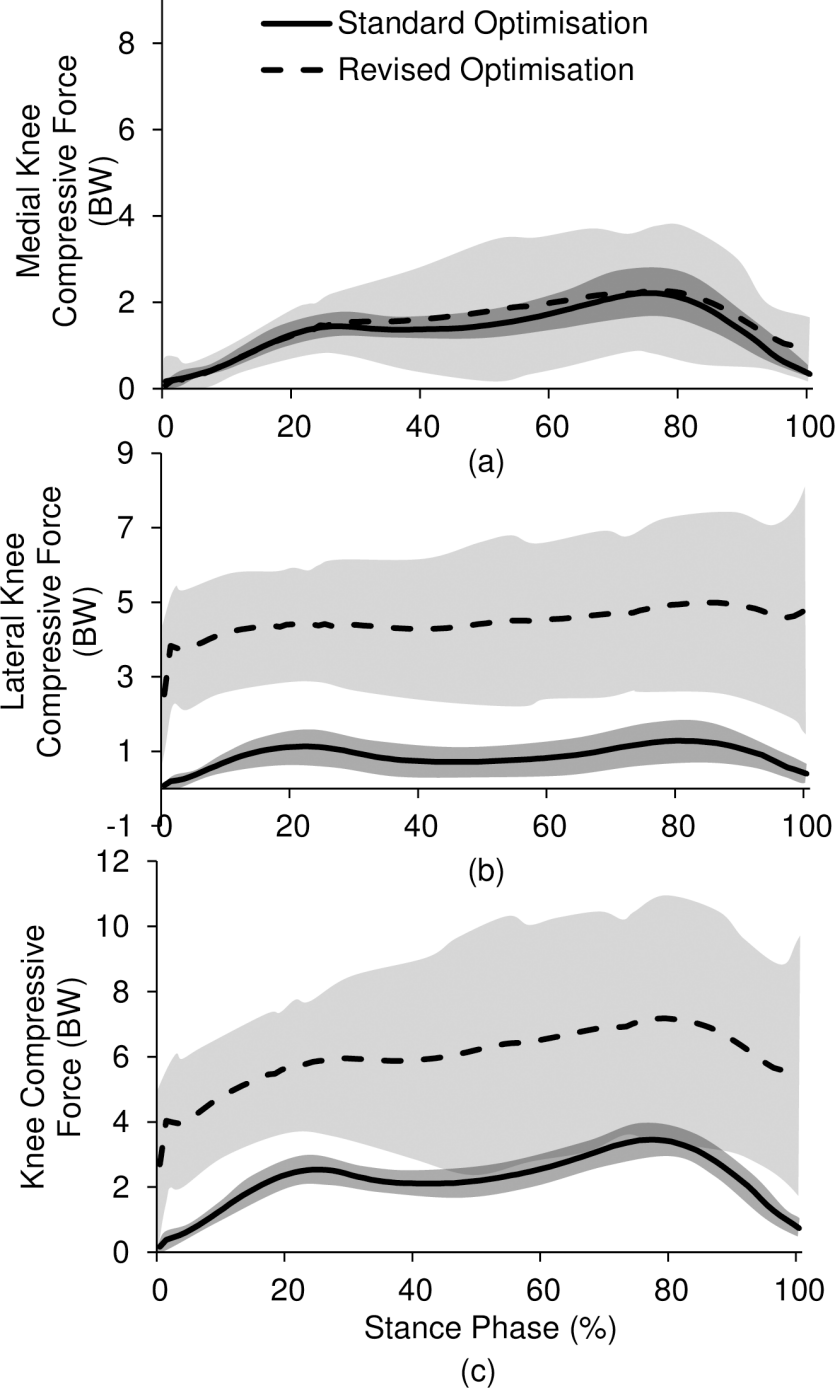
Knee joint compressive forces using standard and revised optimisation: (a) medial, (b) lateral, (c) total force (n = 12).

The knee compressive force with 0.208 BFLH muscle activation is shown in Fig 6. The first peak of lateral knee compressive force was increased by 276% (Fig 6(B), *p*<0.0001) during FES gait when compared to normal gait, resulting in an increase in overall knee compressive force of 144% (Fig 6(C), *p* = 0.0003). There was no significant difference for the first peak of medial knee compressive force (Fig 6(A), *p* = 0.2373).

**Fig 7:**
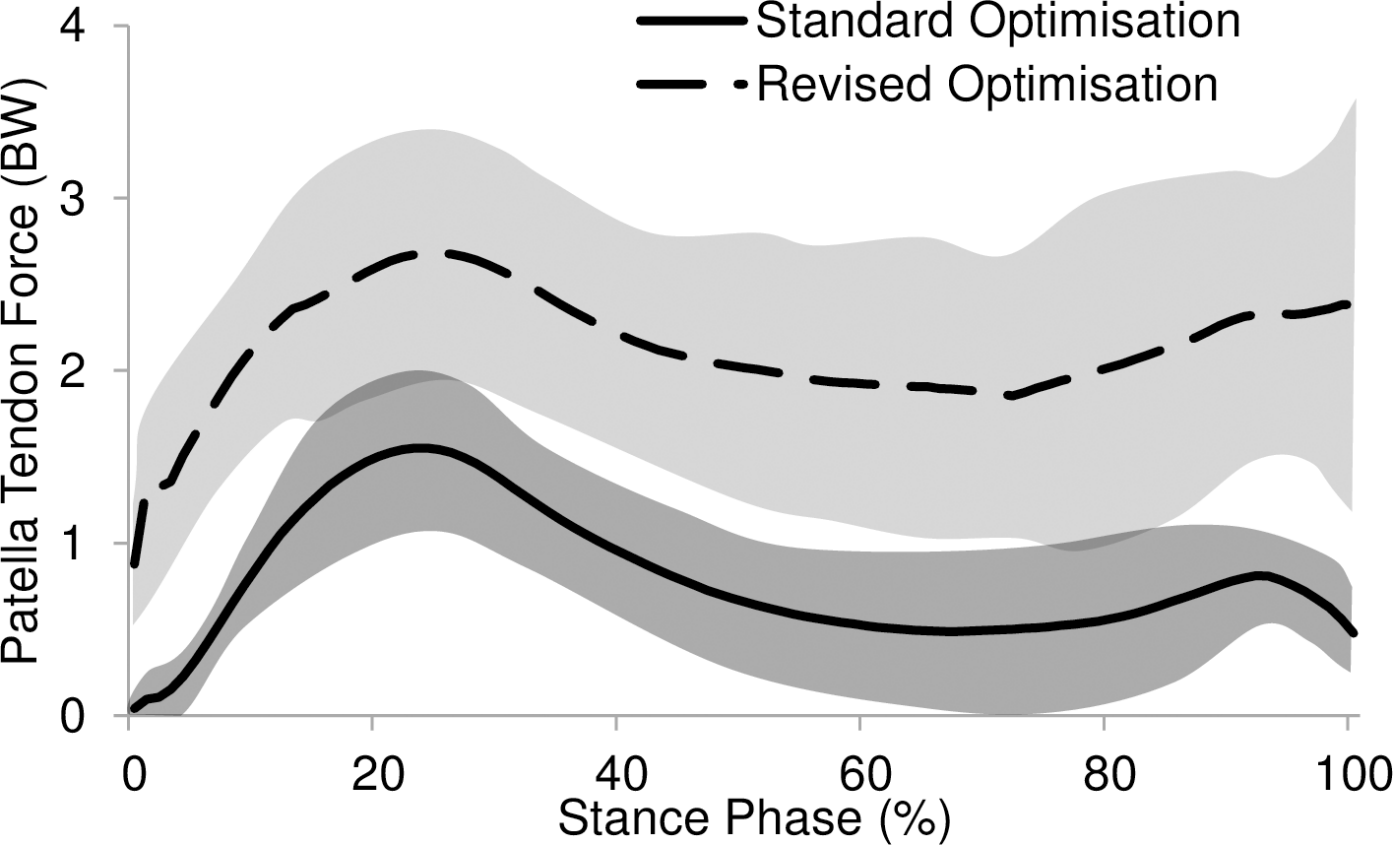
Patella tendon force using standard and revised optimisation (n = 12).

The patella tendon force with 0.208 BFLH muscle activation is shown in Fig 7. The first peak of patella tendon force was increased significantly (*p* = 0.0000) by 61% from 1.5700 ± 0.4635 BW in normal gait to 2.9100 ± 0.7922 BW during the FES gait.

## Discussion

This study tested, using a combined modelling and experimental approach, the hypothesis that selective activation of the BFLH, one of the hamstrings, can theoretically and practically reduce the anterior tibial shear and knee internal rotation torque at the knee. The hypothesis was derived due to the anatomy of the muscle, which attaches on the fibular head that articulates with the lateral tibia and so has the potential to resist a large internal rotation torque and hence the pathological motion of the lateral compartment that occurs in ACL deficiency [5, 6]. We found that the anterior shear force and the knee internal rotation torque were reduced when BFLH was stimulated with FES.

FES gait was slower than healthy gait and therefore it is possible that the reduction in internal rotation torque may be due to this small 7% reduction in speed in addition to that due to the FES assisted muscle activation. However, it is expected that this effect due to speed is small compared to the large 63% reduction in internal rotation torque. Furthermore, the BFLH stimulation does not affect the knee adduction torque and flexion torque (Fig 3(B) and 3(C), p>0.01).

The modelling approach used two optimisation methods to solve the muscle indeterminacy problem; both of these optimisation methods show that when the ACL is loaded during weight acceptance in FES gait the peak tibial internal rotation torque was reduced. The reduction of the tibial internal rotation torque indirectly affects the value of the anterior shear force [32]. Theoretically, as BF inserts on the fibula its activation in a flexed knee is able to pull the tibia posteriorly. In this study, the peak anterior shear force was significantly reduced when FES was applied during weight acceptance, before full knee extension. This work is consistent with the model simulation by Shelburne et al [4] and the experimental study by Chen et al [20] showing that by increasing the muscle activation of the hamstrings, ATT was reduced by 0.2 cm with the knee in 20° to 50° of flexion. Also, in healthy gait body weight is transferred onto the forward limb in the weight acceptance phase. In contrast, for FES gait, the posterior pull of the extra activation of the BFLH by the FES resulting in slower than normal gait, as found in our study.

Here, the modelling cost function was modified from its standard form by assigning a weighting, *c*, to simulate BFLH stimulation. The value of *c* for each subject that reduced anterior shear force to zero was found and the mean value of *c* across all was 0.208. This mean value was then used, resulting in only one subject having a very small positive anterior shear force, demonstrating that the use of a mean value to simulate external activation using FES is appropriate. This particular subject required a *c* value greater than 0.208 to decrease anterior shear force. This may be due to the subject’s characteristics: this was the tallest and heaviest subject. This work also follows the literature in which a similar *c* value of 0.25 was used to simulate the electrically stimulated muscle activation of gluteus medius to reduce the medial knee joint reaction force [33]. In the literature, hamstrings activation without FES has shown that 56% of the maximal hamstring muscle force could reduce the ATT to a normal level during the stance phase of gait [22]. That study modelled motions in the sagittal plane only and so cannot be compared for tibial internal rotation. Focusing on ATT only would suggest that the hamstrings on the medial side could also reduce anterior shear force and this has been shown in other modelling studies [4]. However, as these do not assess tibial internal rotation torque, their results cannot be compared here.

It should be noted that in this study over activation of the hamstrings resulted in a higher knee contact force due to the co-contraction of the quadriceps muscles to overcome the flexion torque due to the hamstrings activation. This has been addressed in a previous study by Catalfamo et al. [14] who proposed that a 50% biceps femoris stimulation is more appropriate than a 100% stimulation to reduce ATT due to the pathological increase in knee joint forces and we have provided further evidence for this proposal.

This study has some limitations. Firstly, the test cohort comprised only healthy control subjects; future work should focus on conducting experiments on ACL deficient subjects to test the applicability of this method in a clinical cohort. We would expect the results in such a cohort to be amplified as an ACL deficient subject would have reduced ability of the passive stabilisers to resist the ATT and internal rotation torque, thus emphasising the effect of the musculature. Secondly, future work should investigate not only the effect of activation of BFLH in ACL deficient subjects, but also include the effect of stimulating other muscles. A confounding factor in ACL deficient subjects is that they already demonstrate altered muscle activation patterns that might result in a different pattern of internal rotation torque and anterior shear force [5, 34, 35]. Thus, such studies might also include an investigation of compensatory muscle activations due to selective activation of BFLH, perhaps through the use of electromyography. Third, the use of static optimisation to determine the muscle forces needs to be further validated, as it may not reflect in vivo muscle force generation [36]. Fourth, the timing of FES can be improved by placing the switch under the subject’s heel, to enable the FES to be set to to high stimulation during heel strike of the injured leg and set to low stimulation during heel strike of the contralateral leg. However, in this study the four seconds of stimulation was enough to make sure the high stimulation occurred during stance phase and was synchronized with the revised optimisation. Finally, the application of a constant muscle activation for the whole of stance phase as achieved here is neither desirable, nor practical. Technology to allow selective activation at the peak of anterior tibial shear should be developed for appropriate clinical use.

## Conclusion

This study is the first to have shown that selective activation of the BFLH can reduce the anterior tibial shear and tibial internal rotation torque at the knee in healthy subjects. It opens the way for new rehabilitation therapies for ACL deficient subjects using FES.

## References

[1] Andersen HN, Dyhre-Poulsen P The anterior cruciate ligament does play a role in controlling axial rotation in the knee. Knee Surg Sports Traumatol Arthrosc. 1997;5145–149. 10.1007/s001670050042 9335025

[2] Noyes FR, Mooar PA, Matthews DS, Butler DL The symptomatic anterior cruciate-deficient knee. Part I: the long-term functional disability in athletically active individuals. J Bone Joint Surg Am. 1983;65(2):154–162. 6687391 10.2106/00004623-198365020-00003

[3] Duthon VB, Barea C, Abrassart S, Fasel JH, Fritschy D, Menetrey J Anatomy of the anterior cruciate ligament. Knee Surg Sports Traumatol Arthrosc. 2006;14(3):204–213. doi: 10.1007/s00167-005-0679-9 16235056 10.1007/s00167-005-0679-9

[4] Shelburne KB, Torry MR, Pandy MG Effect of muscle compensation on knee instability during ACL-deficient gait. Med Sci Sports Exerc. 2005;37(4):642–648. 15809564 10.1249/01.mss.0000158187.79100.48

[5] Gao B, Zheng NN Alterations in three-dimensional joint kinematics of anterior cruciate ligament-deficient and -reconstructed knees during walking. Clin Biomech (Bristol, Avon). 2010;25(3):222–229. 10.1016/j.clinbiomech.2009.11.006 20005613

[6] Amis AA, Bull AMJ, Lie DT Biomechanics of rotational instability and anatomic anterior cruciate ligament reconstruction. Oper Tech Orthop. 2005;15(1):29–35.

[7] Bull AMJ, Andersen HN, Basso O, Targett J, Amis AA Incidence and mechanism of the pivot shift: An in vitro study. Clin Orthop Relat Res. 1999;363219–231. 10379326

[8] Shimokochi Y, Shultz SJ Mechanisms of noncontact anterior cruciate ligament injury. J Athl Train. 2008;43(4):396–408. doi: 10.4085/1062-6050-43.4.396 18668173 10.4085/1062-6050-43.4.396 PMC2474820

[9] Shao Q, MacLeod TD, Manal K, Buchanan TS Estimation of ligament loading and anterior tibial translation in healthy and ACL-deficient knees during gait and the influence of increasing tibial slope using EMG-driven approach. Ann Biomed Eng. 2011;39(1):110–121. doi: 10.1007/s10439-010-0131-2 20683675 10.1007/s10439-010-0131-2 PMC3010217

[10] Noyes FR, Bassett RW, Grood ES, Butler DL Arthroscopy in acute traumatic hemarthrosis of the knee. Incidence of anterior cruciate tears and other injuries. J Bone Joint Surg Am. 1980;62(5):687–695, 757. 7391091

[11] Noyes FR, Matthews DS, Mooar PA, Grood ES The symptomatic anterior-cruciate deficient knee. Part II: the results of rehabilitation, activity modification, and counseling on functional disability. J Bone Joint Surg Am. 1983;65(2):163–174. 6822580 10.2106/00004623-198365020-00004

[12] Solomonow M Sensory-motor control of ligaments and associated neuromuscular disorders. J Electromyogr Kines. 2006;16(6):549–567. 10.1016/j.jelekin.2006.08.004 17045488

[13] Lohmander LS, Englund PM, Dahl LL, Roos EM The long-term consequence of anterior cruciate ligament and meniscus injuries: osteoarthritis. Am J Sports Med. 2007;35(10):1756–1769. doi: 10.1177/0363546507307396 17761605 10.1177/0363546507307396

[14] Catalfamo PF, Aguiar G, Curi J, Braidot A Anterior cruciate ligament injury: Compensation during gait using hamstring muscle activity. Open Biomed Eng J. 2010;499–106. 10.2174/1874120701004010099 PMC2923375 20721326

[15] Escamilla RF, Macleod TD, Wilk KE, Paulos L, Andrews JR Anterior cruciate ligament strain and tensile forces for weight-bearing and non-weight-bearing exercises: a guide to exercise selection. J Orthop Sports Phys Ther. 2012;42(3):208–220. doi: 10.2519/jospt.2012.3768 22387600 10.2519/jospt.2012.3768

[16] Rudolph KS, Axe MJ, Buchanan TS, Scholz JP, Snyder-Mackler L Dynamic stability in the anterior cruciate ligament deficient knee. Knee Surg Sports Traumatol Arthrosc. 2001;9(2):62–71. doi: 10.1007/s001670000166 11354855 10.1007/s001670000166

[17] Sinkjaer T, Arendt-Nielsen L Knee stability and muscle coordination in patients with anterior cruciate ligament injuries: An electromyographic approach. J Electromyogr Kines. 1991;1(3):209–217. 10.1016/1050-6411(91)90036-5 20870511

[18] Andriacchi TP, Dyrby CO Interactions between kinematics and loading during walking for the normal and ACL deficient knee. J Biomech. 2005;38(2):293–298. doi: 10.1016/j.jbiomech.2004.02.010 15598456 10.1016/j.jbiomech.2004.02.010

[19] Lie DT, Bull AMJ, Amis AA Persistence of the mini pivot shift after anatomically placed anterior cruciate ligament reconstruction. Clin Orthop Relat Res. 2007;457 203–209. doi: 10.1097/BLO.0b013e3180314b01 17195812 10.1097/BLO.0b013e3180314b01

[20] Chen CF, Kuo YH, Luh JJ, Chen YJ, Chen SW, Kuo TS, et al Reducing anterior tibial translation by applying functional electrical stimulation in dynamic knee extension exercises: Quantitative results acquired via marker tracking. Clin Biomech (Bristol, Avon). 2013;28(5):549–554. 10.1016/j.clinbiomech.2013.03.007 23583096

[21] Markolf KL, O'Neill G, Jackson SR, McAllister DR Effects of applied quadriceps and hamstrings muscle loads on forces in the anterior and posterior cruciate ligaments. Am J Sports Med. 2004;32(5):1144–1149. doi: 10.1177/0363546503262198 15262635 10.1177/0363546503262198

[22] Liu W, Maitland ME The effect of hamstring muscle compensation for anterior laxity in the ACL-deficient knee during gait. J Biomech. 2000;33(7):871–879. 10831762 10.1016/s0021-9290(00)00047-6

[23] Yanagawa T, Shelburne K, Serpas F, Pandy M Effect of hamstrings muscle action on stability of the ACL-deficient knee in isokinetic extension exercise. Clin Biomech. 2002;17 705–712. 10.1016/s0268-0033(02)00104-3 12446167

[24] Biscarini A, Botti FM, Pettorossi VE Selective contribution of each hamstring muscle to anterior cruciate ligament protection and tibiofemoral joint stability in leg-extension exercise: a simulation study. Eur J Appl Physiol. 2013;113(9):2263–2273. doi: 10.1007/s00421-013-2656-1 23670482 10.1007/s00421-013-2656-1

[25] Hermens HJ, Freriks B, Disselhorst-Klug C, Rau G Development of recommendations for SEMG sensors and sensor placement procedures. J Electromyogr Kines. 2000;10 361–374. 10.1016/s1050-6411(00)00027-4 11018445

[26] Duffell LD, Hope N, McGregor AH Comparison of kinematic and kinetic parameters calculated using a cluster-based model and Vicon's plug-in gait. Proc Inst Mech Eng H. 2014;228(2):206–210. doi: 10.1177/0954411913518747 24449800 10.1177/0954411913518747

[27] Cleather DJ, Bull AMJ The development of a segment-based musculoskeletal model of the lower limb: introducing FreeBody. R Soc Open Sci. 2015;2140449. 10.1098/rsos.140449 PMC4632533 26543569

[28] Ding Z, Nolte D, Tsang CK, Cleather DJ, Kedgley AE, Bull AMJ In vivo knee contact force prediction using patient-specific musculoskeletal geometry in a segment-based computational model. J Biomech Eng. 2016;138(2):021018 doi: 10.1115/1.4032412 26720641 10.1115/1.4032412

[29] Dumas R, Aissaoui R, de Guise JA A 3D generic inverse dynamic method using wrench notation and quaternion algebra. Comput Methods Biomech Biomed Engin. 2004;7(3):159–166. doi: 10.1080/10255840410001727805 15512759 10.1080/10255840410001727805

[30] Crowninshield RD, Brand RA A physiologically based criterion of muscle force prediction in locomotion. J Biomech. 1981;14(11):793–801. 7334039 10.1016/0021-9290(81)90035-x

[31] Yamaguchi GT. Dynamic modeling of musculoskeletal motion: A vectorized approach for biomechanical analysis in three dimensions. New York: Springer; 2001.

[32] More R, Karras B, Neiman R, Fritschy D, Woo S, Daniel D Hamstring an anterior cruciate ligament protagonist. Am J Sports Med. 1993;21(2):231–237. doi: 10.1177/036354659302100212 8465918 10.1177/036354659302100212

[33] Rane L, Bull AMJ Functional electrical stimulation of gluteus medius reduces the medial joint reaction force of the knee during level walking. Arthritis Res Ther. 2016;18(1):255 doi: 10.1186/s13075-016-1155-2 27809923 10.1186/s13075-016-1155-2 PMC5094077

[34] Berchuck M, Andriacchi TP, Bach BR, Reider B Gait adaptations by patients who have a deficient anterior cruciate ligament. J Bone Joint Surg Am. 1990;72(6):871–877. 2365720

[35] Rudolph KS, Eastlak ME, Axe MJ, Snyder-Mackler L 1998 Basmajian Student Award Paper: Movement patterns after anterior cruciate ligament injury: a comparison of patients who compensate well for the injury and those who require operative stabilization. J Electromyogr Kines. 1998;8(6):349–362. 10.1016/s1050-6411(97)00042-4 9840891

[36] Anderson FC, Pandy MG Static and dynamic optimization solutions for gait are practically equivalent. J Biomech. 2001;34153–161. 10.1016/s0021-9290(00)00155-x 11165278

